# Effects of Different Nitrogen Concentrations on Co-Production of Fucoxanthin and Fatty Acids in *Conticribra weissflogii*

**DOI:** 10.3390/md21020106

**Published:** 2023-02-01

**Authors:** Xiangyu Rui, David Kwame Amenorfenyo, Ke Peng, Haoming Li, Linfei Wang, Xianghu Huang, Changling Li, Feng Li

**Affiliations:** College of Fisheries, Guangdong Ocean University, Zhanjiang 524088, China

**Keywords:** *Conticribra weissflogii*, nitrogen concentration, fucoxanthin, fatty acid

## Abstract

Fucoxanthin and fatty acids are active substances that are beneficial to the growth and immunity of humans and aquatic animals. However, relatively few species have been exploited for fucoxanthin and fatty acids in the industry. At the same time, due to its low extract content, poor stability, high production cost, and serious seasonal and regional limitations, the industry cannot normally meet the greater demand of the international market. Therefore, this experiment seeks to improve the fucoxanthin and fatty acid content of *C. weissflogii* by adjusting the nitrogen concentration in the culture medium. It was found that when the nitrogen concentration was 150 mg L^−1^, the cell number was 1.5 × 10^6^ cell mL^−1^, and the average biomass was 0.75 g L^−1^. The mean value of carotenoid concentration was 2.179 mg L^−1^. The average concentration of fucoxanthin was 1.547 mg g^−1^. When the nitrogen concentration was 75 mg L^−1^, the fatty acid content reached its highest. By adjusting the concentration of nitrogen, the contents of fucoxanthin and fatty acids were increased. The results provided a theoretical basis for commercial extraction of fucoxanthin and fatty acids and further promoted the industrialization of fucoxanthin and fatty acids.

## 1. Introduction

Microalgae have a wide distribution, a wide variety, fast growth and reproduction, and contain a variety of biologically active substances [1]. They are biological resources with great potential for development. It is rich in carbohydrates, lipids, proteins, minerals, and other nutrients and can synthesize unsaturated fatty acids (DHA, EPA, etc.), carotenoids (β-carotene, astaxanthin, lutein, Fucoxanthin, etc.), antioxidant substances, active peptides, and other biologically active substances [2,3,4]. Microalgae are widely used in many production fields, such as aquatic biological bait, feed additives, water quality regulation, algal phase construction, water purification, and active substance development, and has an extremely extensive and important application value [5].

Fucoxanthin is a carotenoid widely present in marine microalgae, golden algae, diatoms, and other marine algae. It has many benefits, including antioxidants, weight loss, anticancer, blood sugar regulation, skin protection, and prevention of Alzheimer’s. It is commonly used in the fields of biomedicine, functional food, etc. Microalgae that have been developed and characterized for fucoxanthin synthesis in the past decade are mainly distributed in 20 genera and 49 species, most of which have fucoxanthin content in the range of 1–10 mg/g, while the content of flavin in their extract is low, the stability is poor, the production cost is high, and they are severely limited by seasonal and regional restrictions, so they cannot normally meet the large demand of the international market [6,7,8,9,10].

In aquaculture, diatoms are the most commonly used biological bait for invertebrate aquatic animals. The cells are rich in eicosapentaenoic acid (EPA), docosahexaenoic acid (DHA), and other highly unsaturated fatty acids. Diatoms are also an important potential resource for the large-scale production of EPA and DHA products [11,12]. Numerous studies have shown that EPA and DHA play an important role in nutritional fortification, prevention, and treatment of various diseases, such as heart disease, cancer, inflammation, asthma, diabetes, etc. At present, Japan and the United States have used microalgae to produce PUFAs on a large scale, and the development of PUFAs while using microalgae technology in China is still in the research stage [13].

Marine diatoms are important primary producers in marine ecosystems. In *C. weissflogii,* a typical marine diatom, the intracellular content of fucoxanthin is hundreds of times higher than that of some large brown algae and is considered to be a new source of algae-based fucoxanthin [14,15,16]. *C. weissflogii* is a fast-growing, highly adaptable marine diatom that can grow photosynthetically, autotrophically using CO_2_, and parthenogenetically using an additional nitrogen source. It has been found that the maximum biomass and pigment content can be achieved by using sodium nitrate as a source of nitrogen compared to other nitrogen sources [17,18,19,20]. Most of the research on the culture of Willett’s seaweed has been focused on growth and oil production, with very few reports on the efficient production of luteolin and fatty acids under parthenogenic conditions. This experiment was conducted to study the effects of different nitrogen concentrations on the cell growth, biomass, fucoxanthin, and fatty acid accumulation in the culture conditions of the algae and to screen and optimize the culture medium and culture strategy for the efficient co-production of fucoxanthin and fatty acids in the algae.

## 2. Results

### 2.1. The Growth of C. weissflogii under Different Nitrogen Concentration Culture Conditions

**Figure 1:**
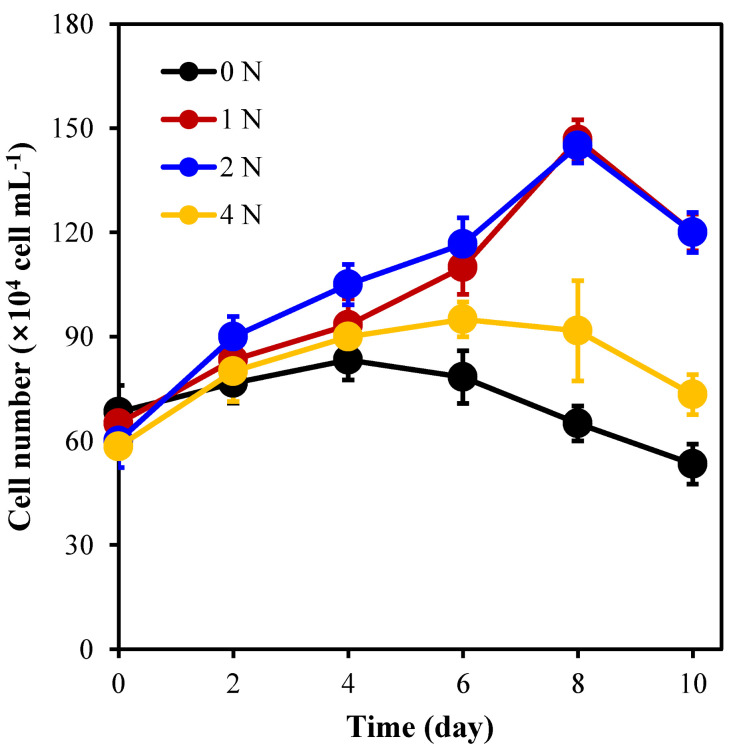
Cell density of *C. weissflogii* under different nitrogen concentration culture conditions.

Figure 1 shows that under the 1 N and 2 N culture conditions, the average algal cell density reached its highest on the 8th day and was significantly higher than that in the 0 N and 4 N groups (*p* < 0.05). It has been proven that nitrogen concentration has a significant effect on the cell growth of *C. weissflogii*. After 8 days of culture, the average density of algal cells in group 2 N reached 1.5 × 10^6^ cell mL^−1^, which was 1.5 times higher than the initial density (6 × 10^5^ cell mL^−1^). It can be seen that under the 2 N culture conditions, the growth state of algal cells is the best, and the growth of the 0 N and 4 N groups is slower.

**Figure 2:**
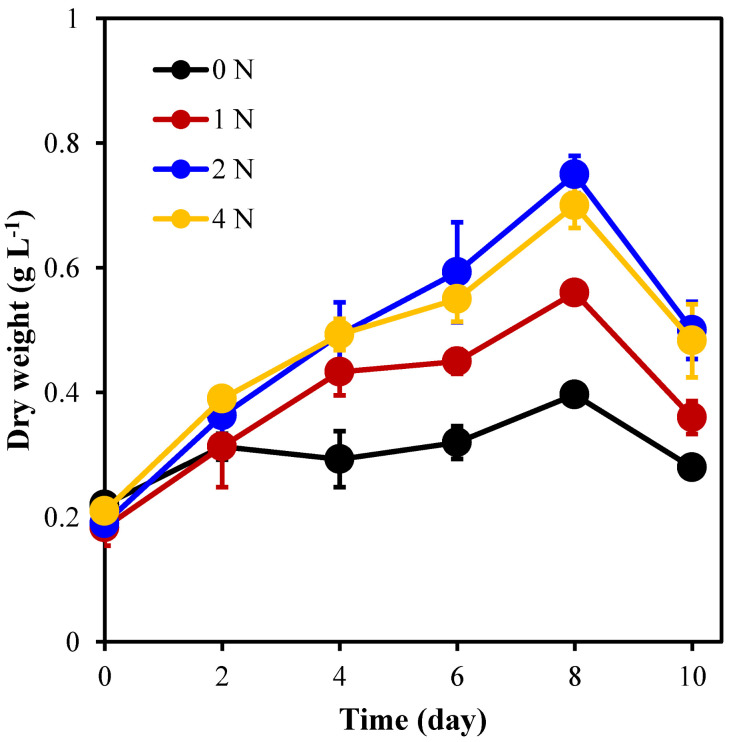
Dry weight of *C. weissflogii* under different nitrogen concentration culture conditions.

It can be seen from Figure 2 that the average algal cell biomass of each group reached its peak on the 8th day; however, the biomass concentration (0.75 g L^−1^) of the 2 N group was significantly (*p* < 0.05) higher than that of the 0 N and 4 N groups with biomass concentrations of 0.5 g L^−1^ and 0.7 g L^−1^, respectively, which was 2.9 times higher than the initial biomass.

### 2.2. The Pigment Content of C. weissflogii under Different Nitrogen Concentration Culture Conditions

**Figure 3:**
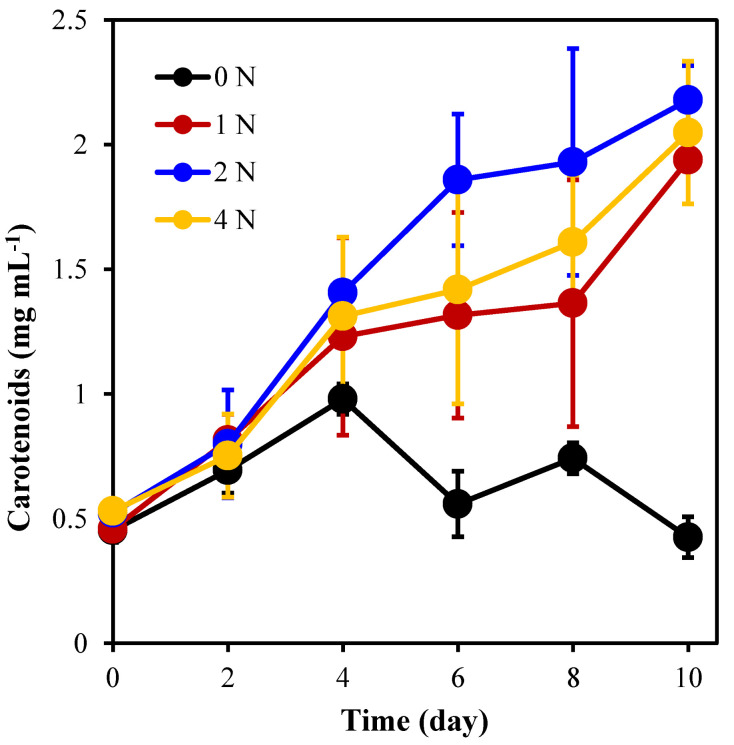
Carotenoid content of *C. weissflogii* under different nitrogen concentration culture conditions.

Figure 3 shows that the nitrogen concentration has a significant effect on the carotenoid content of *C. weissflogii* (*p* < 0.05). During the culture period, except for 0 N, the carotenoid content of the other groups accumulated to different degrees, and the carotene content of the 2 N group was significantly higher than that in the other groups. The average carotenoid concentration in the 2 N group reached 2.179 mg L^−1^ when cultured for 10 days, which was 3.2 times higher than the initial value (0.522 mg L^−1^) and significantly higher than the other three groups. It can be seen that under 2 N culture conditions, algal cells accumulated the highest carotenoid content.

**Figure 4:**
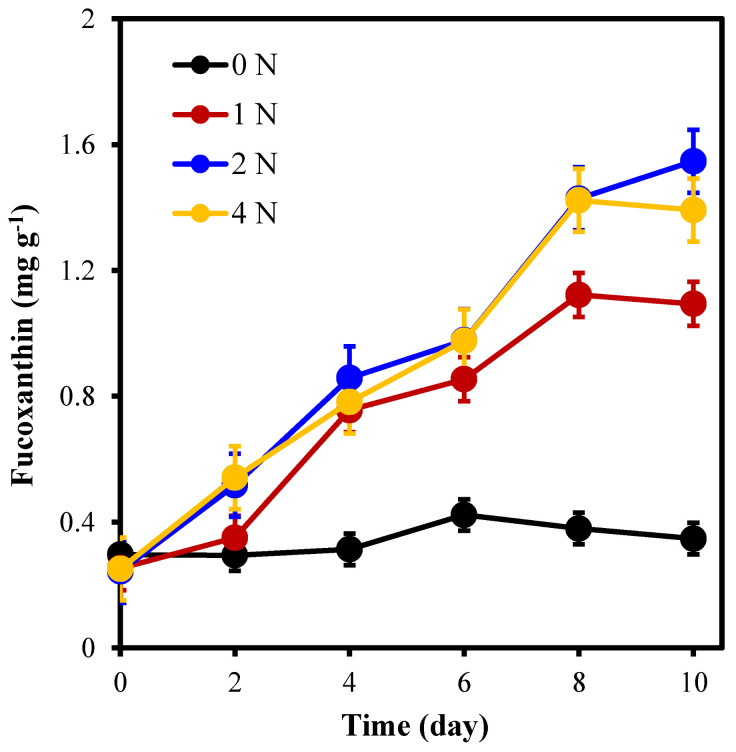
Fucoxanthin content of *C. weissflogii* under different nitrogen concentration culture conditions.

Figure 4 shows that nitrogen concentration had a significant effect on the fucoxanthin content of *C. weissflogii* (*p* < 0.05). During the culture period, the fucoxanthin content of each group accumulated to different degrees, and algal cells in the 2 N group accumulated the highest fucoxanthin content of all groups (*p* < 0.05). The average fucoxanthin concentration was the highest at 1.547 mg g^−1^ when cultured at 2 N for 10 days, which was 2.5 times higher than the initial value (0.244 mg g^−1^) and was 4.5, 1.4, and 1.1 times that of 0 N, 1 N, and 4 N cultures, respectively.

### 2.3. Effects of Different Nitrogen Concentrations on Biomass Productivity and Fucoxanthin Productivity of C. weissflogii

**Figure 5:**
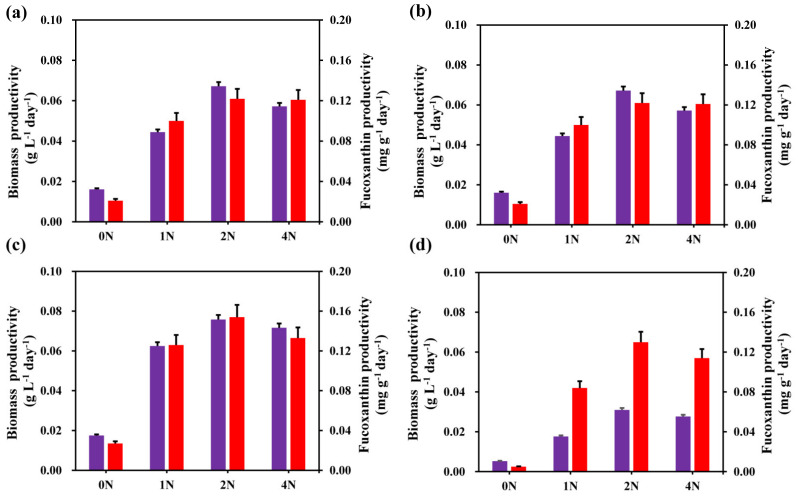
The biomass (purple) and fucoxanthin (red) productivity on day 4 (**a**), day 6 (**b**), day 8 (**c**), and day 10 (**d**).

As shown in Figure 5, the biomass and fucoxanthin productivity of *C. weissflogii* were significantly different under different nitrogen concentrations. We selected the biomass and fucoxanthin productivity on the 4th, 6th, 8th, and 10th days of the experiment for comparative analysis. It can be clearly seen that the maximum biomass productivity of each group occurred in the early stage of cultivation, and the maximum fucoxanthin productivity occurred in the late stage of cultivation. Furthermore, the 2 N group had the highest biomass productivity in each experimental group. The highest fucoxanthin productivity in each experimental group was also in the 2 N group, except for on the 6th day. Therefore, we determined that the 2 N culture conditions were more suitable for biomass and fucoxanthin accumulation in *C. weissflogii*.

### 2.4. Fatty Acid Composition and Content of C. weissflogii under Different Nitrogen Concentrations

As can be seen from Table 1, a total of 29 fatty acids were detected under different nitrogen concentration culture conditions, including 15 saturated fatty acids (SFA) and 14 unsaturated fatty acids (UFA) (4 monounsaturated fatty acid (MUFA) and 5 polyunsaturated fatty acid (PUFA)). The main fatty acid species of this algae are C16:ln7, C16:0, C20:5n3, C22:6n3, and C15:0. Among them, the total content of UFA was significantly higher than that of SFA, and there were differences under different nitrogen concentration culture conditions. The order of fatty acid content from high to low was SFA (41.0–43.0%) > MUFA (36.7–43.0%) > PUFA (14.7–21.9%).

**Figure 6:**
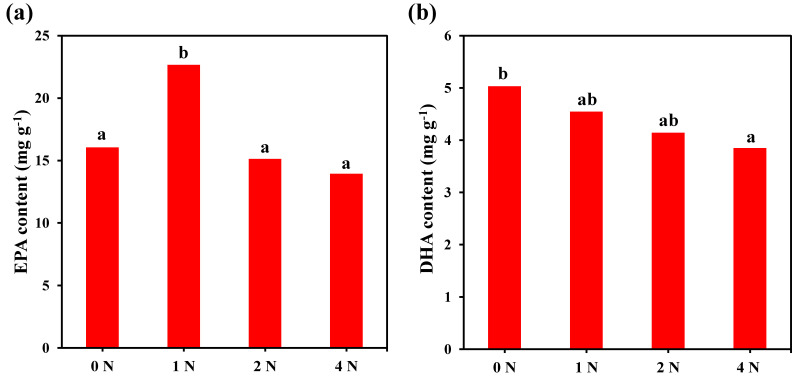
EPA (**a**) and DHA (**b**) content of *C. weissflogii* under different nitrogen concentration culture conditions. Means within the same column of different letters are significantly different at (*p* < 0.05).

The content of EPA under 1 N culture conditions was significantly higher than that of other culture conditions, among which C16:ln7, C16:0, EPA, and DHA changed significantly compared with other fatty acids, which were significantly affected by changes in algae cell culture conditions. Under 1 N culture conditions, the content of SFA, EPA, and total fatty acids in the algae cells was the highest, which was significantly higher than that of other experimental groups (*p* < 0.05). It can be seen from Figure 6 that the nitrogen concentration has a significant effect on EPA, and the content of EPA is the highest in the 1 N cultures; its EPA content is about 22.7 mg g^−1^, accounting for 10.6% of the total content. We also found that the nitrogen concentration had a significant effect on the DHA content, which showed that the DHA content gradually decreased with the increase of the nitrogen concentration during the culture process, and the highest content was about 5.0 mg g^−1^ under the 0 N condition, accounting for a total fatty acid content of up to 3.3% with different levels of changes in EPA and total fatty acid content.

In a large number of studies based on diatom fatty acids, it is also shown that almost all diatoms contain C16:0, C16:ln7, EPA, and DHA, with some species of C15:0, C16:3(n-4) content also being high. While most C18 and C22 diatoms have lower PUFA content, the results of this experiment are basically consistent with those in the literature.

## 3. Discussion

Nitrogen is the basic element that microalgae use to synthesize pigments, proteins, and nucleic acids and accounts for 1−10% of the dry weight of microalgae cells. It is one of the most important nutritional factors that limit the growth of microalgae cells and plays an extremely important role in the growth and metabolism of algae [21]. Different forms and concentrations of nitrogen have different effects on the growth, pigmentation, and lipid accumulation of microalgae [22]. He Sisi et al. [23] used different concentrations of sodium nitrate to culture *Eustigmatos vischeri.* Their results showed that a higher biomass concentration of 9.14 g L^−1^ yielded 17.6 mmol L^−1^ nitrogen concentrations, but with low total lipid content. However, at lower (3.0 mmol L^−1^) nitrogen concentrations, the lipid content gradually increased, reaching a maximum of 60.82 %. Wang et al. [24] used *Platymonas subcordiformis* as the test material to investigate the effects of different sodium nitrate concentrations on the growth of *Platymonas subcordiformis* and the content of oil and starch. The results showed that at a 10 mg L^−1^ sodium nitrate concentration, *P. subcordiformis* experienced slow growth with low biomass concentration; however, the oil and starch content of *P. subcordiformis* were higher at the same sodium nitrate level. When the mass concentration of sodium nitrate was 60 mg L^−1^, the oil and starch content were higher, the growth of *Platymonas* was faster, and the overall total yield of oil and starch was higher. When the concentration of sodium nitrate continued to increase, the biomass and content of each component were basically unchanged. Zou et al. [25] discovered higher growth and biomass concentration (5.19 × 10^5^ cells mL^−1^) of *Haematococcus pluvialis* when the concentration of sodium nitrate was 0.15 g L^−1^. This is similar to the present study, even though a different source of nitrogen was used. In our study, a higher growth rate and biomass concentration were obtained at a nitrogen concentration of 150 mg L^−1^ which could serve as the optimum nitrogen concentration for maximum growth and biomass production of *C. weissflogii*.

Furthermore, assimilation of various nutrients in the culture medium could lead to the production of various intracellular products, which could affect the content of biomass, pigments, and fatty acids [26,27]. Since different nitrogen concentrations have different accumulation conditions for various substances [28,29], the findings show that the changes in algal biomass were different from the changes in algal cell density.

The difference in nitrogen concentration will not only affect the growth rate of microalgae cells, but also affect the synthesis and accumulation of substances in the microalgae cells [30]. In particular, the pigment content has a great influence. In the absence of nitrogen addition, the carotenoid content decreases rapidly, as does the fucoxanthin content [31]. At the same time, too high a nitrogen concentration will also limit the growth of the microalgae, thereby affecting the carotenoid and fucoxanthin content [32,33]. Only under a suitable nitrogen concentration will pigments effectively accumulate in the microalgae, and the carotenoid and fucoxanthin content will gradually increase over an increasing culture period [34]. During the cultivation process of this experiment, we found that the carotenoid and fucoxanthin contents in the algal cells of the other experimental groups had accumulated to different degrees, except for those without nitrogen addition. Too high or too low of a nitrogen concentration will indeed affect the accumulation of carotenoid and fucoxanthin. However, with an increase in culture time, the nitrogen in the medium will be consumed, the growth of microalgae will be limited, and the content of carotenoid and fucoxanthin will gradually decrease. These results are consistent with those of Carreto and Catoggio, who found that carotenoid and fucoxanthin contents first increased and then decreased with the increase in *Phaeodactylum tricornutum* culture time [35]. Changing nutrient composition can cause stress in the physiological activity of algae, which can trigger dramatic changes in the growth of microalgae and their carotenoid production [36,37].

Fatty acids are the main components of lipids in algal cells, which can provide energy sources for algae in adversity and have important physiological functions. Liang et al. [38] found that changes in nitrogen concentration had a significant effect on the fatty acid composition of microalgae. Studies have shown that the fatty acid content of *Nannochloropsis oculata* increases with an increase in nitrogen concentration and that both PUFA and EPA levels show an upward trend [39]. In general, the ratio of PUFA and EPA in microalgae increases with increasing nitrogen concentration [40]. However, not all algae follow this trend, and there is a green alga that synthesizes more PUFAs under nitrogen starvation conditions, indicating that the effect of nitrogen concentration on the fatty acid composition of microalgae varies from species to species [41,42]. This study also confirmed this conclusion, as increasing nitrogen concentrations resulted in decreasing PUFA and DHA contents in *C. weissflogii*. The highest levels were achieved without nitrogen addition, but the total fatty acid content and EPA content were different. They reach a maximum value at a nitrogen concentration of 75 mg L^−1^ and decrease with further increases in nitrogen concentration. The effect of nitrogen concentration on the algae was demonstrated to promote the accumulation of PUFAs under unfavorable growth conditions. In addition, Liu Yun et al. [43] found that when the nitrogen concentration was between 0 and 25 μmol L^−1^, the fatty acid content was significantly higher than that of other groups, indicating that nitrogen limitation was beneficial to the accumulation of fatty acids. This may be related to the upregulation of key enzymes involved in fatty acid synthesis under nitrogen-limited conditions [44] since most PUFAs are synthesized by fatty acid elongase and desaturase with SFA as the substrate. Therefore, nitrogen limitation may reduce the activity of these enzymes, resulting in the accumulation of SFA and a decrease in PUFA content [45].

## 4. Materials and Methods

### 4.1. Experimental Instruments and Materials

**Figure 7:**
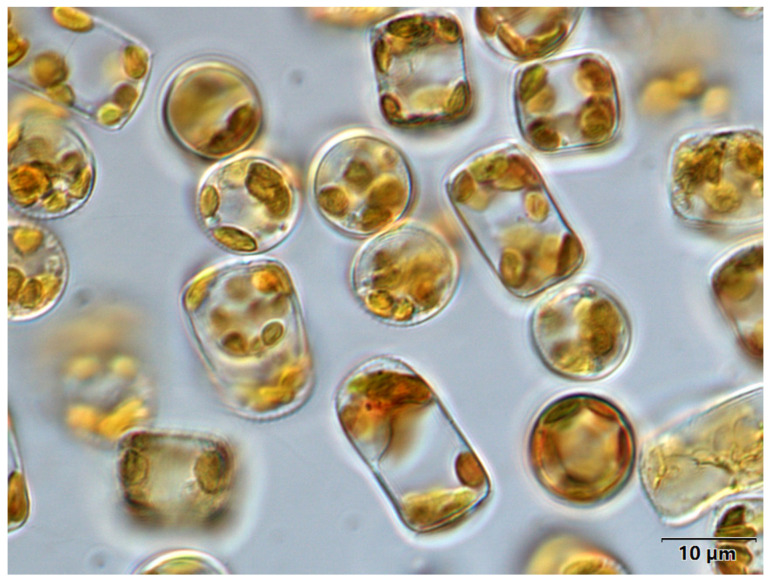
Optical microscope image of *C. weissflogii* used in this study.

The algae (Figure 7) used in the experiment was isolated and purified from shrimp ponds in southern China by the Laboratory of Algal Resources Development and Ecological Remediation of Aquaculture Environment, Guangdong Ocean University. Centrifuged and concentrated algal sap from the index growth period was used for the experiment. The main experimental equipment includes a columnar photo bioreactor, a Shanghai Yaoxun intelligent autoclave, a Japan Shimadzu uv2450 ultraviolet spectrophotometer (Shimadzu Corporation, Tokyo, Japan), a Japan Olympus BX53 fluorescence microscope blood cell counting plate (Olympus Corporation, Tokyo, Japan), a Tianjin Zinteng GM-0.33A vacuum pump (Zingteng Experimental Equipment Co., Ltd., Tianjin, China), a Beijing Sihuan freeze dryer (Foring Technology Developmen Co., Ltd., Beijing, China), a Multifuge high-speed frozen centrifuge, a Shanghai Boxun GZX-9140MBE electric heating blast drying oven (Boxun Medical Bioinstrument Co., Ltd., Shanghai, China), and an inflatable pump. Cells were incubated in a continuous light aerated sealed culture using modified F/2 medium.

A modified version of the F/2 medium was used, with NaNO_3_ 75 mg, KH_2_PO_4_ 5mg, Na_2_SiO_3_-9H_2_O 20 mg, F/2 trace element solution 1 mL, and F/2 vitamin solution 1 mL per liter of double-distilled water. F/2 Trace element solution formulation is as follows: C_10_H_14_N_2_Na_2_O_8_ 4160 mg, FeC_6_H_5_O_7_ 3150 mg, MnCl_2_·4H_2_O 180 mg, ZnSO_4_·4H_2_O 22 mg, CuSO_4_·5H_2_O 10 mg H_4_MoNa_2_O_6_ 6 mg, and CoCl_2_·6H_2_O 4160 mg per liter of double distilled water. F/2 vitamin solution is formulated with Biotin 0.5 mg, Vitamin B_12_ 0.5 mg, and Vitamin B_1_ 100 mg per liter of double-distilled water.

### 4.2. Algae Strain and Culture Conditions

The F/2 nitrogen-deficient medium was prepared to use sodium nitrate as the nitrogen nutrient source, with a gradient of 0 N, 1 N, 2 N, and 4 N (0 mg L^−1^, 75 mg L^−1^, 150 mg L^−1^ and 300 mg L^−1^), a volume of 700 mL, an initial inoculum density of approximately 6 × 10^5^ cells mL^−1^. The experiments were conducted under continuous light, aeration, temperature of 25 ± 2 °C, illumination level of 30 ± 2 µmol m^−2^ s^−1^, pH 8.0 ± 0.2, and salinity of 25, with three parallel incubation periods of 10 d for each treatment. Samples taken every two days.

### 4.3. Analytical Methods

The cell density of *C. weissflogii* was counted under a BX53 fluorescence microscope using a Blood Counting Plate (25 mm × 16 mm) after each sampling.

Biomass was determined using the drying differential weight method (measurement the 1um acetate membrane which was baked to a constant mass and recorded as mass M_1)_. 10 mL of algal liquid °C in a centrifuge tube was then filtered with a vacuum pump with discardement of the filtrate, washed and centrifuged twice with distilled water, placed the filter membrane in an oven, dried at 80 °C to a constant mass, measured, and recorded the total mass M_2_. The dry weight (DW) was calculated with the following Formula (1). Each sample was set up in three parallels, averaged, and the standard deviation calculated.

(1) $$\mathrm{DW}=\left(M_{2}-M_{1}\right)/10$$

The content of carotenoids was determined by the ethanol extraction method [46]. 10 mL of algal liquid was centrifuged at 5000 r/min for 10 min, the supernatant was discarded, 10 mL of ethanol was added with a volume fraction of 95%, dark-treated for 24 h, centrifuged at 5000 r/min for 10 min, and the supernatant was measured using a spectrophotometer The optical density value (D) of the solution was measured at 480, 510, and 750 nm. The content of carotenoids was calculated according to the following formula (2) [47].

(2) $$\rho \left(\mathrm{Carotenoids}\right)=7.6\times \left[\left(D_{480}-D_{750}\right)-1.49\times \left(D_{510}-D_{750}\right)\right]$$

In the formula, D_480_, D_510_, and D_750_ are the optical densities at 480, 510, and 750 nm, respectively.

Fucoxanthin with extracted using organic solvents [48]. 80 mL of cultured *C. weissflogii* was centrifuged at 5000 r min^−1^ for 10 min at 4 °C, the supernatant was discarded, freeze-dried for 2 d, and grind into powder. The algal flour was added to absolute ethanol to make the ratio of material to liquid 1 g: 40 mL, and the extraction was performed at 60 °C in the dark, and extracted twice for 1h each time. After leaching, the algal liquid was centrifuged at 5000 r min^−1^ for 10 min, the supernatant was taken, and the absorbance (D_445_) was measured at 445 nm using an ultraviolet spectrophotometer. The fucoxanthin content C can be expressed as:

(3) $$C\left(\mathrm{Fucoxanthin}\right)=\left(1000\times {\;D}_{445}\times \;N\;\times \;V\right)/\left(\;A^{\prime }\times \;M\;\times 100\right)$$

In the formula: C is the content of fucoxanthin, the unit is mg/g; D_445_ is the absorbance of fucoxanthin at 445 nm; N is the dilution ratio; V is the volume of the crude extract; A′ is the theoretical absorption value of a solute with a volume fraction of 1% in a colorimetric cup of 1 cm optical range length and has a value of 1600; M is the sample mass.

Fatty acid composition was determined by gas chromatography [49]. An appropriate amount of sample was weighed into a glass tube, 0.5 mol L^−1^ NaOH methanol solution was added and then shocked evenly at 60 °C water bath for 20 min, saponified and cooling; added boron trifluoride methanol complexing solution and shocked evenly at 60 °C for 6 min for methylation; after cooling, isooctane was added for extraction, filtered with a 0.45 nm organic filter membrane, and the supernatant was taken into the injection bottle for determination.

The prepared samples were analyzed by an Agilent 7890A gas chromatograph. The parameters were: capillary column (DB-23MS, column length 60 m, inner diameter 0.25 mm, film thickness 0.15 μm); split injection mode, split ratio 35:1; carrier gas is nitrogen; inlet temperature 270 °C, initial temperature 100 °C, lasting for 13 min; 100 °C~180 °C at a heating rate of 10 °C min^−1^ for 6 min; 180 °C~200 °C at a heating rate of 1 °C min^−1^ for 20 min; 200 °C~230 °C at a heating rate of 4 °C min^−1^ for 10.5 min; detector: FID. Automatic injection: 1 μL sample solution was injected into the gas chromatograph, and the retention time and peak height of the chromatographic peak were recorded. The retention time of each chromatographic peak was determined by the standard chromatogram, and the percentage of each fatty acid component was calculated by the automatic integral method of the software.

Sensitivity analysis was performed to check the robustness of fatty acid content.

## 5. Conclusions

In this work, we examined the effect of different nitrogen concentrations on the production of the carotenoid pigment fucoxanthin and fatty acids by the marine microalgae *C. weissflogii*. It was found that when the nitrogen concentration was 150 mg L^−1^, the yield of fucoxanthin was highest on the 10 th day. The average concentration of fucoxanthin was 1.547 mg g^−1^, which was 5.3 times higher than the initial value (0.244 mg g^−1^). At 75 mg L^−1^, the fatty acid content reached the maximum, the EPA content was about 22.7 mg g^−1^, accounting for 10.6% of the total fatty acid content, and the PUFA and DHA content reached the maximum when no nitrogen was added. These concentrations are higher than those of other species of microalgae reported in the literature, highlighting the potential of *C. weissflogii* as a source of fucoxanthin and fatty acids. The results of this work provide a systematic study of the effect of nitrogen concentration on fucoxanthin production from *C. weissflogii*. These data have obvious applications in the design and scale-up of processes for fucoxanthin production.

## Figures and Tables

**Table 1:** Fatty acid composition and content (μg/g dry biomass) of *C. weissflogii* under different nitrogen concentration culture conditions.

Test Items	0 N	1 N	2 N	4 N
C4:0	510.09	72.78	72.43	95.07
C8:0	451.62	205.67	111.82	144.97
C10:0	837.58	91.67	749.12	897.8
C11:0	205	146.07	80.59	103.76
C12:0	845.07	312.87	128.85	152.21
C14:0	202.17	19,855.7	12,896.25	11,319.31
C14:1n5	Not detected	248.02	185.78	172.25
C15:0	7911.41	3760.83	2158.12	1680.39
C16:0	44,175.05	62,837.67	45,565.71	36,811.21
C16:1n7	53,077.27	86,394.56	64,680	55,224.8
C17:0	1385.3	672.74	443.44	478.44
C18:0	1222.82	779.43	496.11	441.97
C18:1n9c	1640.05	1953.58	1160.62	875.18
C18:2n6c	1378.37	212.06	571.51	555.39
C18:3n6	641.67	238.42	246.95	315.25
C18:3n3	Not detected	165.81	260.83	468.74
C20:0	799.48	212.06	106.15	145.1
C20:2	2113.57	238.42	91.63	212.83
C20:3n6	383.46	165.81	148.27	271.33
C21:0	1090.76	124.29	96.37	473.16
C20:4n6	905.97	1729.42	768.33	1204.2
C20:5n3	16,058.6	22,672.09	15,140.51	13,953.13
C22:0	109.23	412.22	347.82	332.29
C22:1n9	511.66	82.83	232.45	139.27
C22:2n6	6725.25	2653.1	1155.3	997.14
C23:0	1369.49	809.51	753.65	569.1
C24:0	1651.71	1292.15	833.15	853.62
C22:6n3	5035.53	4550.28	4146.78	3852.98
C24:1n9	501.59	134.97	103.68	98.63
SFA	62,766.78	91,585.66	64,839.58	54,498.4
MUFA	55,730.57	88,813.96	66,362.53	56,510.13
PUFA	33,242.42	32,625.41	22,530.11	21,830.99
FA	151,739.8	213,025	153,732.2	132,839.5
